# Relationship between mitochondrial haplogroup and physiological responses to hypobaric hypoxia

**DOI:** 10.1186/s40101-016-0094-6

**Published:** 2016-04-29

**Authors:** Midori Motoi, Takayuki Nishimura, Yuka Egashira, Fumi Kishida, Shigeki Watanuki

**Affiliations:** 1 Faculty of Design, Kyushu University, 4-9-1 Shiobaru, Minamiku, Fukuoka 815-8540 Japan; 2 Department of Public Health, Nagasaki University Graduate School of Biomedical Science, 1-12-4, Sakamoto, Nagasaki-shi, Nagasaki 852-8523 Japan; 3 Graduate School of Integrated Frontier Sciences, Kyushu University, 4-9-1 Shiobaru, Minamiku, Fukuoka 815-8540 Japan

**Keywords:** Mitochondrial haplogroup, Hypobaric hypoxia, SpO_2_

## Abstract

We aimed to investigate the relationship between mtDNA polymorphism and physiological responses to hypobaric hypoxia. The study included 28 healthy male students, consisting of 18 students in haplogroup D and 10 in haplogroup M7+G. Measurement sensors were attached to the participants for approximately 30 min in an environment with a temperature of 28 °C. After resting for 15 min, the programmed operation of the hypobaric chamber decreased the atmospheric pressure by 11.9 Torr every minute to simulate an increase in altitude of 150 m until 9.7 Torr (equivalent to 2500 m) and then decreased 9.7 Torr every minute until 465 Torr (equivalent to 4000 m). At each altitude, the pressure was maintained for 15 min and various measurements were taken. Haplogroup D showed higher SpO_2_ (*p* < 0.05) and significantly higher SpO_2_ during the pressure recovery period when compared with haplogroup M7+G. The distal skin temperature was higher in haplogroup D when compared with M7+G. These results suggested that haplogroup D maintained SpO_2_ at a higher level with higher peripheral blood flow during acute hypobaric exposure.

## Introduction

Approximately 100,000 years ago, humans left Africa, spreading across the world and adapting to various environments [1]. This history of human migration is frequently assessed by mitochondrial-DNA (mtDNA) analysis. Previous studies have suggested that not only is mtDNA evolutionarily neutral but it is also the cause for natural selection against specific environmental pressures, such as cold and high altitude [2, 3]. Consistent with that, we have reported the relationship between physiological responses to cold and mtDNA haplotype [4, 5]. Moreover, mtDNA haplotype might also be related to high-altitude adaptation [6]. Hypoxia is characterized by a lack of oxygen in relation to aerobic adenosine triphosphate (ATP) requirements and increased reactive oxygen species (ROS) generation. The common mtDNA haplotypes determine differences in oxidative phosphorylation (OXPHOS) performance and ROS production in both mice and humans [7–9].

Peripheral artery oxygen saturation (SpO_2_) is a useful index to indicate the physiological status in high-altitude environments*.* Because of the decrease in atmospheric pressure at high altitudes, SpO_2_ becomes diminished. Typically, maintaining high SpO_2_ is important in preventing acute mountain sickness (AMS) [10]; however, differences exist between different populations and between individuals. Differences in oxygen saturation at the population level are being researched currently. Beall [11] reported that Tibetans tend to have lower SaO_2_ compared with Andean highland natives. Additionally, Weitz et al. [12] reported no differences between Han migrants and Tibetans, but some studies have reported that Han individuals are more desaturated than Tibetans during sleep [13] and exercise [14–16]*.* Within populations, individual variations in SpO_2_ levels increase at higher altitudes, indicating a physiological polytypism in SpO_2_ in hypobaric hypoxia. Although exposure to hypobaric hypoxia is limited in lowlanders, this variation may include genetic factors*.* Interestingly, Li et al. [17] reported a relationship between mtDNA polymorphism and the risk of developing AMS. They subjectively evaluated AMS in the Han Chinese population using the Lake Louise Self-Assessment Score and suggested that there was a low risk of developing AMS in haplogroups D and M9 but a high risk in haplogroups G and M7. The AMS-tolerant haplogroups D and M9 were frequent among those who actually lived in the Tibetan highlands [18].

Although the relationship between mtDNA polymorphism and high-altitude adaptation has been reported [6, 17], there have been no studies on the relationship between mtDNA polymorphism and the physiological state at high altitudes. Therefore, in the present study, we aimed to investigate the relationship between mtDNA polymorphism and physiological responses to hypobaric hypoxia. Because haplogroup D is AMS-tolerant and is common among the Tibetan population, we hypothesize that haplogroup D will respond better to hypobaric exposure when compared to the other group.

## Methods

### Study participants

The study included 28 healthy Japanese male students (aged 19–24 years) who had neither heart nor ear diseases. Medium- and long-term highland residents within 2 months were not included. Participants consisted of 18 students in haplogroup D and 10 in haplogroup M7+G, which are from the same M-type lineage. Table 1 shows the basal metabolic rate and physical data (height, weight, and body mass index) for the groups. The number of subjects who exercised more than three times a week was 10 in the D group and 4 in the M7+G group. The number of smokers (defined as smoking more than one cigarette per day) was four in the D group and one in the M7+G group. There was no significant difference between groups. Subjects were prohibited from taking exercise and drinking alcohol 1 day prior to the experiment and from eating, smoking, and drinking caffeine for 2 h before the exercise. The study procedure was thoroughly explained, and the students participated in the study after providing written consent.

**Table 1:** Mean (±SE) body measurements in haplogroups D and M7+G

Group	Height (cm)	Weight (kg)	BMI (kg/m^2^)	BMR (ml/kg/min)
D (*n* = 18)	172.2 (0.01)	64.6 (3.56)	21.8 (1.18)	3.90 (0.09)
M7+G (*n* = 10)	170.5 (0.02)	60.5 (3.58)	20.8 (1.23)	3.75 (0.19)

*BMI* body mass index, *BMR* basal metabolic rate

### Study protocol

**Fig. 1:**
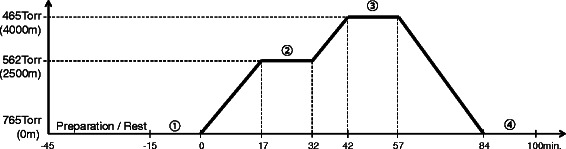
Experimental protocol

Study participants were asked to refrain from eating and drinking 2 h prior to the beginning of the study. They wore t-shirts and shorts, and the experiment was conducted while they rested while seated in a chair. The itinerary of the experiment is shown in Fig. 1. Various measurement sensors were attached to the participants for approximately 30 min while the temperature was maintained at 28 °C; the participants subsequently entered the hypobaric chamber. After resting quietly for 15 min, the programmed operation of the hypobaric chamber decreased the atmospheric pressure by 11.9 Torr every minute to simulate an increase in altitude of 150 m until 562 Torr (equivalent to 2500 m) and then decreased by 9.7 Torr every minute until 465 Torr (equivalent to 4000 m). At each altitude, the pressure was maintained for 15 min and various measurements were taken. Pressure recovery was also performed at an increase of 11.1 Torr per minute to the ambient atmospheric pressure. DNA analysis was performed by the same method as our previous studies [4, 5].

This protocol of hypoxic exposure and mtDNA analysis was performed with approval from the Ethics Committee for Genome-gene Analysis of the Graduate School of Medicine, Kyushu University.

### Measurement parameters

Rectal and skin temperature measurements were sampled at 10-s intervals using a data logger (LT-8A, Gram Corporation, Saitama, Japan). Skin temperature sensors were attached at the forehead, forearm, back of the hand, abdomen, thigh, lower leg, and dorsal side of the foot. Mean skin temperature was calculated by the Hardy-DuBois seven-point method [19].

Distal skin temperature (${\overline{T}}_{\mathrm{dist}}$) was derived using the following equation:

$$ {\overline{T}}_{\mathrm{dist}}=\left(0.14\times {\mathrm{T}}_{\mathrm{arm}}+0.05\times {\mathrm{T}}_{\mathrm{hand}}+0.07\times {\mathrm{T}}_{\mathrm{feet}}+0.13\times {\mathrm{T}}_{\mathrm{leg}}\right)/0.39. $$

SpO_2_ and heart rate (HR) measurements were sampled at 1-min intervals using a Pulse Oximeter Radical-7TM (Masimo Corporation, Tokyo, Japan). Exhaled gas (Douglas bag method) was collected four times at steady state atmospheric pressure at each altitude (pre 0 m, 2500 m, 4000 m, post 0 m). VE was measured with a wet gas meter (W-NK-10A, Shinagawa Corporation, Tokyo, Japan) and calculated by the formula (1):

$$ {\mathrm{VE}}_{\mathrm{B}\mathrm{TPS}}={\mathrm{VE}}_{\mathrm{ATPS}}\times \frac{P_{\mathrm{B}}-{P}_{{\mathrm{H}}_2\mathrm{O}}}{P_{\mathrm{B}}-47}\times \frac{273+37}{273+T} $$

The O_2_ and CO_2_ concentrations in the expired gas were measured with a respiratory gas analyzer (AE-300S, Minato Medical Science, Osaka, Japan). These data were calculated by the formula (2):

$$ {\mathrm{VE}}_{\mathrm{STPD}}={\mathrm{VE}}_{\mathrm{ATPS}}\times \frac{P_{\mathrm{B}}-{P}_{{\mathrm{H}}_2\mathrm{O}}}{760}\times \frac{273}{273+T} $$

The respiratory exchange ratio (*R*) is defined as the ratio of VCO_2_ to VO_2_.

The expired gas concentration was measured with a respiratory gas analyzer (AE-300S, Minato Medical Science, Osaka, Japan). The exhaled gas data of three subjects were not available because of equipment failure. Height, weight, and basal metabolic rate were measured, and a lifestyle survey was conducted to determine the physiological characteristics of the individual.

### Statistical analysis

Data were analyzed by two-way ANOVA with haplogroup and time (altitude) as factors at the 5 % significance level. For multiple comparisons, an unpaired *t* test was performed. All data are given as means ± standard error.

## Results

Physical characteristics did not differ between the groups (Table 1).

### Oxygen saturation (SpO_2_)

**Fig. 2:**
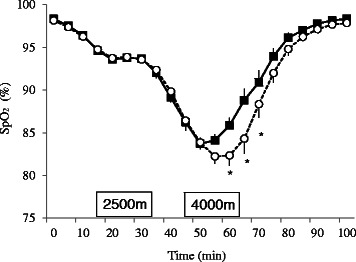
Changes in SpO_2_ (means ± standard error) in haplogroups D (*black squares*; *n* = 18) and M7+G (*white circle*; *n* = 10). ^*^ *p* < 0.05 comparing D and M7+G. SpO_2_ at 60–75 min after the beginning of the experiment was significantly higher in haplogroup D than in haplogroup M7+G subjects

Time had a significant effect (*F*_(20, 520)_ = 117.99, *p* < 0.001), and the interaction between time and group (*F*_(20, 520)_ = 2.112, *p* < 0.005) was also significant (Fig. 2). Post hoc test results revealed that 60–75 min after the beginning of the experiment, SpO_2_ was significantly elevated in haplogroup D when compared with M7+G (*p* < 0.05).

### Distal skin temperature

**Fig. 3:**
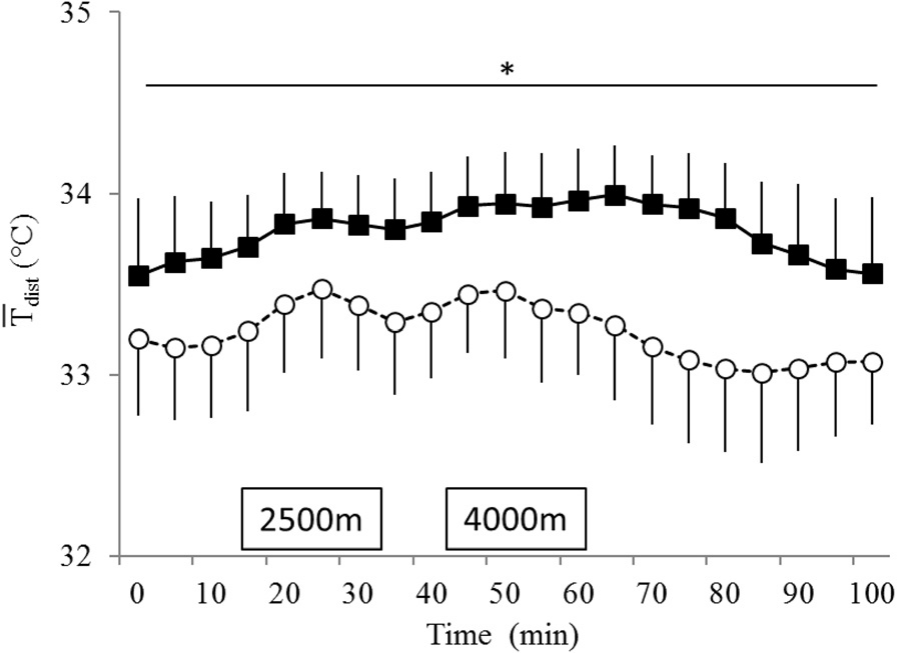
Changes in distal skin temperature (means ± standard error) in haplogroups D (*black squares*; *n* = 18) and M7+G (*white circle*; *n* = 10). Haplogroup D had a significantly higher ${\overline{T}}_{\mathrm{dist}}$ compared to that of haplogroup M7+G (*p* < 0.05) throughout the experiment

Distal skin temperature differed significantly between groups (*F*_(1, 26)_ = 18.51, *p* < 0.001) and over time (*F*_(20, 520)_ = 16.08, *p* < 0.001) (Fig. 3). The interaction between group and time was also significant (*F*_(20, 520)_ = 4.76, *p* < 0.001). The post hoc test results revealed that haplogroup D had a significantly higher ${\overline{T}}_{\mathrm{dist}}$ throughout the experiment when compared with haplogroup M7+G (*p* < 0.05).

### Heart rate

**Fig. 4:**
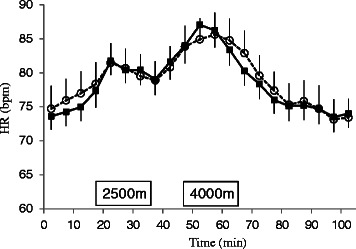
Changes in heart rate (means ± standard error) in haplogroups D (*black squares*; *n* = 18) and M7+G (*white circle*; *n* = 10). Heart rate changed significantly over time and was significantly elevated at 15–70 min compared with at 0 min

There were no significant differences in heart rate between the two groups (*F*_(1, 26)_ = 0.01, *p* = 0.916); however, time did have a significant effect on this parameter (*F*_(20, 520)_ = 26.59, *p* < 0.001) (Fig. 4). Post hoc test results revealed that heart rate was significantly elevated after 15–70 min compared with at 0 min. The interaction between group and time was not significant (*F*_(20, 520)_ = 0.53, *p* = 0.956).

### Minute ventilation $\left(\overset{.}{\mathrm{V}}\mathrm{E}\right)$ and ***R***

**Fig. 5:**
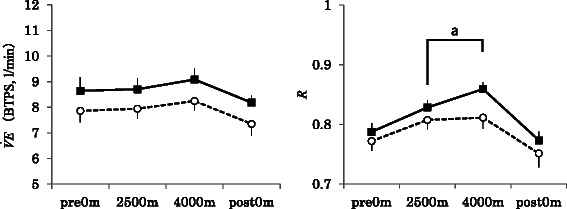
Changes in minute ventilation ($\overset{.}{\mathrm{V}}\mathrm{E}$) and *R* (means ± standard error) in haplogroups D (*black squares*; *n* = 16) and M7+G (*white circle*; *n* = 9). There was no effect of group, time, or their interaction on $\overset{.}{\mathrm{V}}\mathrm{E}$. *R* changed significantly over time. *R* was significantly higher at 4000 m than at 2500 m in haplogroup D, but not in M7+G

There was no significant effect of group, time, or their interaction on $\overset{.}{\mathrm{V}}\mathrm{E}$ (group: *F*_(1, 23)_ = 2.01, *p* = 0.169; time: *F*_(1, 23)_ = 2.53, *p* = 0.064; interaction: *F*_(1, 23)_ = 1.65, *p* = 0.920) (Fig. 5).

There was no significant difference in VO_2_ and VCO_2_ between the two groups (data not shown). *R* did not differ significantly between the two groups (*F*_(1, 23)_ = 1.29, *p* = 0.268), but time was significant (*F*_(3, 69)_ = 21.54, *p* < 0.001). Post hoc test results revealed that *R* was significantly higher at 4000 m than 2500 m in haplogroup D. The interaction between group and time was not significant (*F*_(3, 69)_ = 0.95, *p* = 0.423).

## Discussion

In the present study, oxygen saturation (SpO_2_) at a moderate hypobaric exposure of 2500 m varied very little (Fig. 2). However, at a severe hypobaric exposure of 4000 m, the variation in SpO_2_ increased. In particular, haplogroup D showed higher SpO_2_ (*p* < 0.05) and significantly higher SpO_2_ during pressure recovery (60–75 min) when compared with haplogroup M7+G. These results suggest that haplogroup D can maintain SpO_2_ at a higher level during acute hypobaric exposure and support the results of Li et al. [17]. Previous studies suggested that the advantage of haplotype D is related against oxidative damage because of mtSNP-related amino acid substitutions on mitochondrial NADH dehydrogenase subunit [20]. This anti-oxidative effect may prevent deterioration of hemoglobin function. Thus, individuals with haplogroup D could keep a higher SpO_2_ level than M7+G.

In this study, distal skin temperature was higher in haplogroup D than M7+G (Fig. 3). Haplogroup D exhibited an increase in *R* at 4000 m (Fig. 5), which indicates accentuation of glycolytic metabolism.

The data suggested the possibility of oxygen dissociation curve (ODC) deviation to the right in haplogroup D because the factors that cause this are an increase in carbon dioxide partial pressure PCO_2_, blood temperature, and 2,3-DPG concentration, which is an intermediate product of glycolytic metabolism.

The deviation to the right of the ODC makes it easy to dissociate oxygen in the periphery, leading to improved oxygen supply to the tissues. Therefore, we presume that D group’s O_2_ metabolic efficiency was an adaptive reaction; however, there was no difference in VCO_2_. Therefore, there are two possible explanations: First, deviation to the right of the ODC occurred over a short time; previous studies have reported that this occurred within ~60 min when lowland inhabitants went to high altitudes [21]. Second, haplogroup D might already deviate to the right of the ODC. Even when SpO_2_ is maintained at high levels during hypobaric exposure, if the blood flow is high, long-term adaptation to oxidative stress is unfavorable because CMS occurs. Long-term hypobaric exposure studies are needed to investigate this paradox. Additionally, genetic background, such as EPAS1 and EGLN1 genes, may affect physiological responses [22, 23], and investigation of other specific genes is also necessary. EPAS1 and EGLN1, which are hypoxia-inducible factors (HIF), are genetic mutations specific to the Tibetans. These mutations contribute to suppressing an increase in Hb concentration and cause AMS, which may directly affect high-altitude adaptation. We cannot clarify this here because of the following limitations: First, we could not measure hematocrits and circulation. Thus, we have no data on blood oxygen content. Second, we could not conduct quantitative screening for previous altitude exposure.

In conclusion, SpO_2_ was significantly higher in haplogroup D during extreme hypobaric hypoxia and recovery compared with haplogroup M7+G. However, the underlying mechanism remains unclear, and further study is needed to explain the individual differences in the physiological responses to hypobaric hypoxia.
